# Oleuropein inhibits invasion of squamous cell carcinoma of the head and neck through TGF-β1 signaling pathway

**DOI:** 10.1186/s12885-022-09979-2

**Published:** 2022-09-01

**Authors:** Ting Xu, Xuan Liu

**Affiliations:** Department of Otolaryngology, Wuxi Second Clinical Medical College of Nantong University, No. 68, Zhongshan Road, Liangxi District, Wuxi, Jiangsu, 214002 People’s Republic of China

**Keywords:** Oleuropein, SCCHN, Epithelial-mesenchymal transition, TGF-β1, Metastasis

## Abstract

**Background:**

Squamous cell carcinoma of the head and neck (SCCHN) is globally the sixth most common cancer. TGF-β1 is a key regulator of cell proliferation and differentiation, and it induces the epithelial-mesenchymal transition (EMT) by activating Smad2 signaling in SCCHN cells. Previous studies have revealed that oleuropein (OL) can inhibit the EMT alterations and migration of cancer cells. The aim of this study was to examine the involvement of TGF-β1 signaling pathway in SCCHN and the effect of OL on it.

**Methods:**

Through in vitro experiments at cellular level and in vivo evaluation in mouse xenograft tumor model, with morphological and Western blotting assays, we examined the effects of OL on TGF-β1-mediated signaling pathway in Tu686, CAL-27 and 686LN-M2 tumor cell lines.

**Results:**

We found that OL reversed the TGF-β1-induced EMT, and changed the morphology of cells and the expression levels of epithelial and interstitial markers. Wound-healing and transwell invasion assays indicated that OL reversed the TGF-β1-promoted cell migration and invasion dramatically. The effects of OL were also verified in xenograft tumor model of mice, and the findings were identical to the in vitro assays.

**Conclusion:**

This study demonstrated that OL inhibits the growth and metastasis of SCCHN by interfering with the TGF-β1 signaling pathway, and the findings are beneficial for the development of prevention and treatment strategy of SCCHN. Due to the low toxicity and less side effects, OL may be of potential value in the inhibition of metastasis of SCCHN and improve survival.

**Supplementary Information:**

The online version contains supplementary material available at 10.1186/s12885-022-09979-2.

## Background

Squamous cell carcinoma of the head and neck (SCCHN) is the sixth most common cancer worldwide [1]. Appropriately, 550, 000 cases were newly diagnosed each year, and 300, 000 deaths were linked to SCCHN. Metastasis is a leading cause to the poor prognosis of the disease [2]. The underlying mechanisms of metastasis is a complicated process, but epithelial-mesenchymal transition (EMT) was deemed to be one of the main pathways leading to tumor invasion and metastasis [3]. EMT is a biological process with regard to cell transformation from epithelial state to interstitial state and acquisition of migration and invasion. Previous studies showed that EMT was associated with self-renewal traits, and the promotion of tumor initiation, apoptosis and therapeutic resistance [4]. Moreover, EMT is related to the activation of Smad2/3-dependent signaling pathway mediated by TGF-β1 [5].

TGF-β1 is a key regulator of cell proliferation and differentiation, and belongs to the differentiation β superfamily cytokines. It can not only activate the phenotype transformation of normal fibroblasts, but also stimulates mesenchymal original cells [6]. Furthermore, TGF-β1-induced EMT is mainly mediated by either Smad-dependent or Smad-independent pathways. It was reported that TGF-β1 induced the EMT process by activating Smad2 signal in SCCHN cell line Tu686 [7]. Thus, the activation of anti-EMT pathway, or the inhibition of TGF-β1 signaling, was considered to provide a potentially novel target for the treatment of SCCHN.

Oleuropein (OL), a natural antioxidant molecule extracted from olive leaves, has been reported to be associated with the beneficial effects of the plant on human [8–10]. Studies revealed that OL can suppress the EMT alterations of peritoneal cells during peritoneal dialysis, so as to alleviate the toxicity reaction of dialysis [11]. In addition, it was reported that OL inhibits the migration through suppression of EMT in breast cancer cells [12], and it also inhibits the proliferation of colorectal cancer cell through downregulation of HIF-1α signal [13]. HIF-1α-regulated TGF-β1 can induce EMT to promote the progression of breast cancer [14], and induction of EMT via Wnt/β-catenin signaling has been shown to be driven by HIF-1α in prostate and hepatocellular carcinoma [15, 16]. In this study, we demonstrated that OL inhibits the growth and metastasis of SCCHN by interfering with the TGF-β1 signaling pathway. These results will play a guiding role in the prevention and treatment of SCCHN.

##  Materials and methods

### Cell culture and treatment

The human SCCHN cell lines, Tu686, 686LN-M2 (M2, the corresponding high metastasis potential cell line of Tu686) and CAL-27 (Shanghai NuoChen Biotechnology Co., Ltd), were cultured in 10% fetal bovine serum (FBS) and Dulbecco’s Modified Eagle Medium (DMEM) F12 medium (Gibco, Grand Island, NY, USA), 100 IU/mL penicillin and 100 IU/mL streptomycin at 37 ˚C in a humidified atmosphere with 5% CO_2_. The appropriate treatment concentration of TGF-β1 (recombinant human TGF-β1, Peprotech, USA) was 10 ng/mL according to previous study [7]. OL (CAS no. 10596–60-1, molecular weight 540.514, Shanghai Yeyuan Biology) powder, was resuspended by precooled sterile PBS, with a final concentration of 1 mg/mL (stored at -20 ˚C), and diluted with PBS and the appropriate concentration was selected by cell viability assay. The morphological changes of cells were observed under an inverted fluorescence microscope (Leica DMI3000B, German).

### MTT assay

Cell viability was detected with the 3-(4,5-dimethyl-thiazol-2-yl)-2,5 diphenyltetrazolium (MTT) assay. Tu686 and CAL-27 cells (1 × 10^4^ cells per well) were cultured in 96-well plates. The cells were adhered to the wall on the next day and were starved in serum-free medium overnight. Then the cells were exposed to preset concentrations of OL. After another 24 h, 150 μL of MTT (2 μg/mL; Sigma-Aldrich) was added for incubation at 37 ˚C. Formazan crystals were dissolved in DMSO and the absorbance was measured at 570 nm using a Beckman Coulter microplate reader.

### Apoptosis assay

Cells were inoculated into six-well plates in a density of 1 × 10^5^ cells/well. The next day, the medium was replaced after 5 h of TGF-β1 (10 ng/mL) and/or OL (25 μg/mL) treatment. After another 24 h, the cells were collected, centrifuged at 200 g and resuspended. Cells were then stained by Annexin V-FITC according to the protocol of Annexin V-PI apoptosis detection kit (Kaiji Biology, Jiangsu). Twenty min later, propid iumiodide (PI) was added for another 5 min. Then the cells were analyzed by flow cytometry.

### Scratch and invasion assay

Tu686 and CAL-27 cells were seeded onto each well of the six-well plates (2 × 10^5^ cells per well) and allowed to grow to 90% confluence, then they were placed into serum-free medium for 24 h. The cells were scratched with a 200-µL pipette tip and the non-adherent cells were washed off with medium. Cells were then treated with PBS, OL (25 μg/mL), TGF-β1 (10 ng/mL), and TGF-β1 (10 ng/mL) + OL (25 μg/mL). Migration of wounded cells was observed and photographed at 0 and 24 h with a microscope previously described. Three different areas in each assay were chosen to measure the distance of migrating cells. For invasion assay, 3 × 10^4^ Tu686 or CAL-27 cells in 100 µL of serum-free medium, treated with 10 ng/mL TGF-β1, 25 μg/mL OL, or with 10 ng/mL TGF-βl + 25 μg/mL OL, were seeded in the upper chamber of Matrigel coated inserts (8 µm pores; BD Bioscience). Following incubation for 24 h, the cells which penetrated the filters were stained with gentian violet. The number of invasive cells was determined by counting all cells attached to the bottom of the inserts under an inverted microscope at × 100 magnification. Both assays were carried out in triplicate.

### Western blotting

The assay was performed as previously described [17]. Briefly, a total of 50 µg proteins were extracted from the cells or tissues of each group. After electrophoresis, transmembrane and blocking, the blotted membranes were incubated with anti-E-cadherin (ab40772, abcam, 1:500), anti-Vimentin (ab92547, abcam, 1:500), anti-Snail (ab216347, abcam, 1:1,000), anti-Smad2 (ab280888, abcam, 1:1000), anti-p-Smad2 (3140, Cell Signaling, 1:500), anti-Smad4 (ab40759, abcam, 1:1000), anti-MMP9 (3852, Cell Signaling, 1:1000), anti-N-Cadherin (ab76011, abcam, 1:1000), anti-HIF-1α (ab1, abcam, 1:1000), anti-AKT (9271, Cell Signaling, 1:500), anti-p-AKT (9271, Cell Signaling, 1:500), anti-PHD2 (3293, Cell Signaling, 1:1000) antibodies at 4 ˚C overnight. Subsequent to being washed, the membranes were incubated with HRP-labeled secondary antibodies (Boster Biological Engineering, Wuhan) for 1 h at room temperature. Bands were visualized by employing the BeyoECL Plus Detection system (Beyotime Institute of Biotechnology, Jiangsu, China). The intensity of protein fragments was quantified with Quantity One software (4.5.0 Basic; Bio-Rad, Hercules, CA, USA) and represented as the densitometric ratio of the targeted proteins to β-actin. All cell protein lysates were assayed in triplicate.

### Xenograft tumor experiments

Male, 5 to 7-week-old BALB/c nude mice were purchased from Changzhou Cavens Animal Experiment Company and raised in SPF animal experiment center. Ten mice were divided into control and OL treatment groups. Preparation of cells for transplantation injection: the M2 cells for control group and OL group were treated with PBS and 1200 µg/mL of OL, respectively, after incubation for 24 h, the cells were collected. On the day of inoculation, tumor cells at 70%-80% confluence were trypsinized and resuspended in FBS-free culture medium. A volume of 100 µl single cell suspension was injected into the subcutaneous area of mice, and whether there were colliculus and redness in the injection area was observed. After inoculation, they were kept in SPF animal room. The lengths and widths of the tumors were measured with vernier calipers, and tumor volumes were calculated using the following formula: tumor volume = length × width^2^ × 0.5. Two weeks later, the mice were sacrificed and the tissues were obtained for Western blotting assay. The use of animals in the present study was complied with the Guide for the Care and Use of Laboratory Animals. This study was approved by the Ethics Committee of Wuxi Second People’s Hospital. The approval number is (2021) Ethical Review No. (Y-13). All methods were reported in accordance with the ARRIVE guidelines. The tissue sections were viewed at × 100 magnification, and images were captured with a digital camera.

### Statistical analysis

The statistical analysis software package (SPSS 11.5, Inc., Chicago, IL, USA) was employed for data analysis. Data were expressed as “mean ± SEM”. The Student’s t-test and Mann–Whitney U test were used for the statistical analysis of data. Difference at *P* < 0.05 was considered to indicate a statistical significance.

## Results

### Selection of suitable dose of OL by MTT assay

**Fig. 1:**
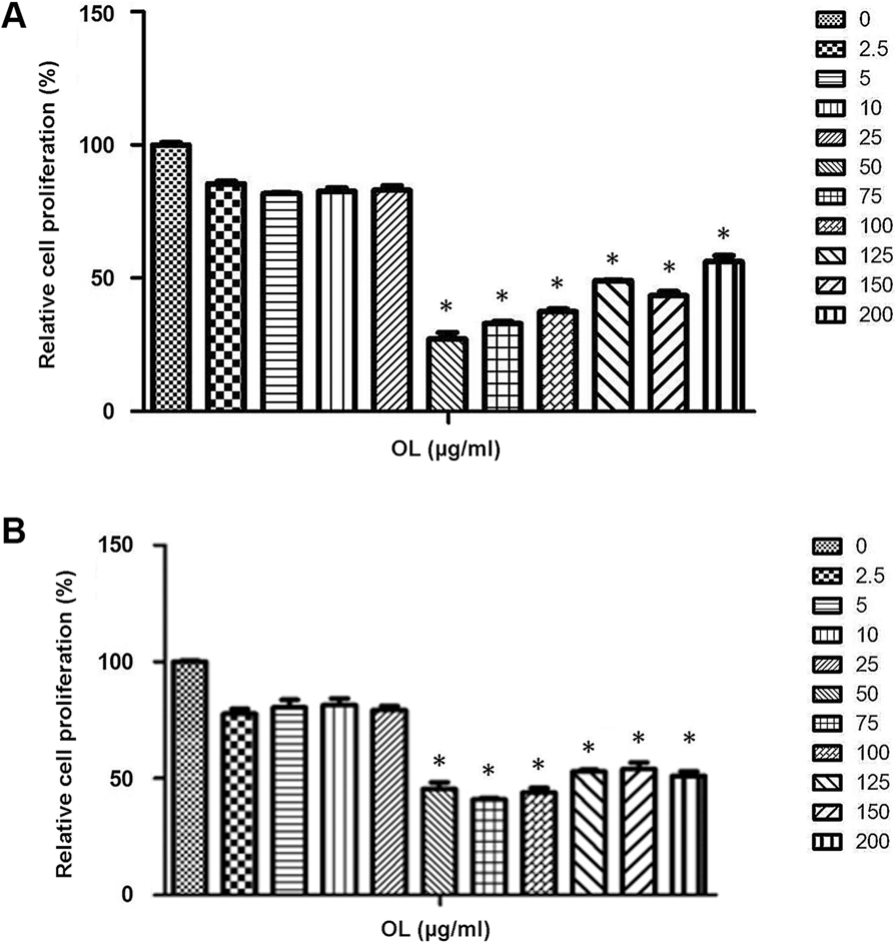
Effect of OL on proliferation of Tu686 and CAL-27 cells. **A** MTT assay was performed in Tu686 cells to determine non-toxic dose range. Cells untreated or treated with increasing doses of OL (range from 2.5-200 µg/mL) for 24 h. Cell proliferation was expressed as fold change with respect to untreated control cells (0 µg/mL). *p <0.05 vs. untreated cells. **B** MTT assay with CAL-27 cells using increasing doses of OL (range from 2.5-200 µg/mL) for 24 h. Cell proliferation was expressed as fold change to untreated cells. *p <0.05 vs. untreated cells

The intervention concentration of OL was set to 0, 2.5, 5, 10, 25, 50, 75, 100, 125, 150, and 200 µg/mL, respectively. By analyzing the cell proliferation activity, we found that it had no side effects on the proliferation of the Tu686 cells under the concentration of 25 µg/mL (Fig. 1A; *n* = 6–9 for each group). But OL given at higher doses from 50 to 200 µg/mL had comparable inhibitory effect on cell proliferation when compared with the control (0 µg/mL). Thus, OL at a concentration of 25 µg/mL was selected as the using intervention dose in vitro. Similar effects of OL were found in cell proliferation experiment with another human SCCHN cell line CAL-27 (Fig. 1B; *n* = 6–9 for each group).

### OL promotes apoptosis of Tu686 and CAL-27 cells

**Fig. 2:**
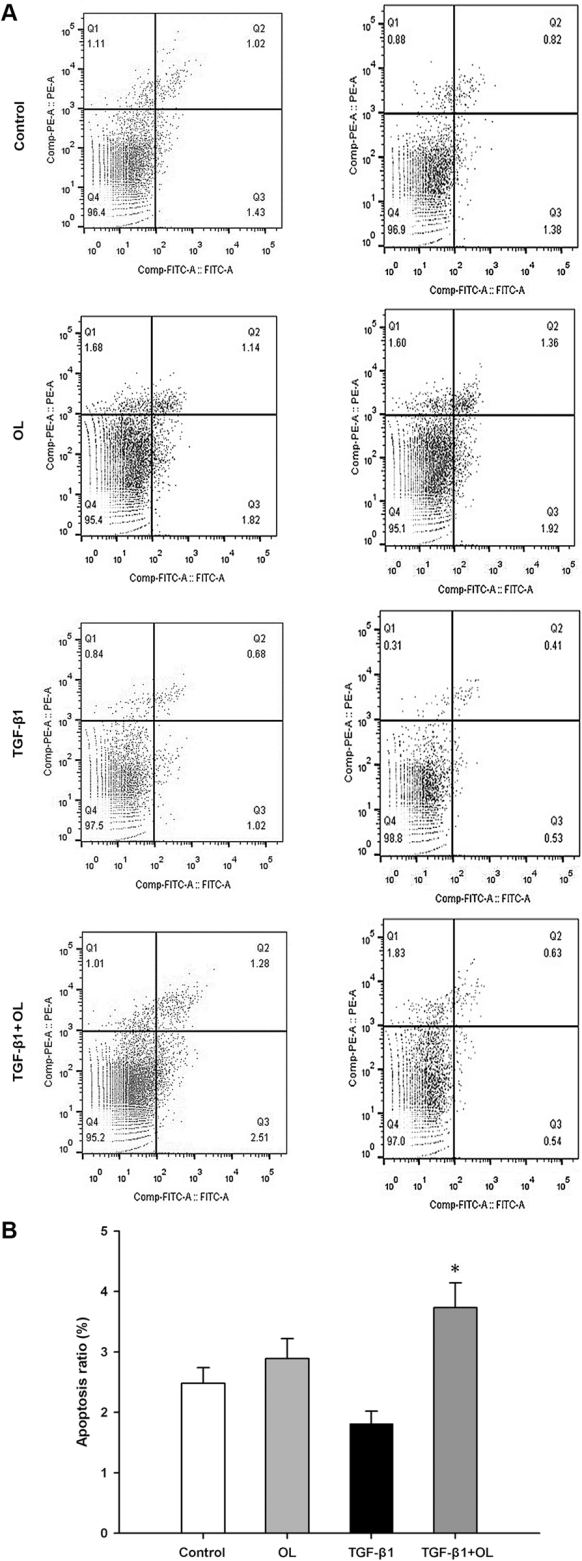
Effect of OL on apoptosis of Tu686 and CAL-27 cells. **A** Flow cytometry apoptosis diagrams for different treatment groups with Tu686 (left lane) and CAL-27 (right lane) cells. The dose of 25 µg/mL of OL was selected for flow cytometry. The cells treated with PBS, 25 µg/mL OL, 10 ng/mL TGF-β1, and 10 ng/mL TGF-β1 + 25 µg/mL OL, were named control, OL, TGF-β1, and TGF-β1 + OL groups, respectively. **B** Comparison column chart of the apoptosis ratio of the four groups with Tu686 cells. Total apoptosis ratio (including early and late) was detected for each group. *p <0.05 vs. control or TGF-β1 group (tests were repeated for 3 times)

The number of Q2 + Q3 cells was obtained from Apoptosis FACS data and utilized for the evaluation of Tu686 cell apoptosis. After TGF-β1 intervention, the number of apoptotic cells decreased slightly without statistically significant difference compared with the control group. After adding both TGF-β1 and OL for intervention, the apoptosis ratio of Tu686 cells increased significantly when compared with that of the control and TGF-β1 alone group (*P* < 0.05), while OL alone had no significant influence on apoptosis ratio (Fig. 2A (left lane) and 2B; *n* = 6 for each group). Moreover, intervention with OL and TGF-β1 on CAL-27 cells for apoptosis assay was also conducted, and the results showed similar trends with the Tu686 cells but without obvious significance (Fig. 2A, down lane; *n* = 3 for each group).

### OL reverses TGF-β1-induced EMT of Tu686 and CAL-27 cells

**Fig. 3:**
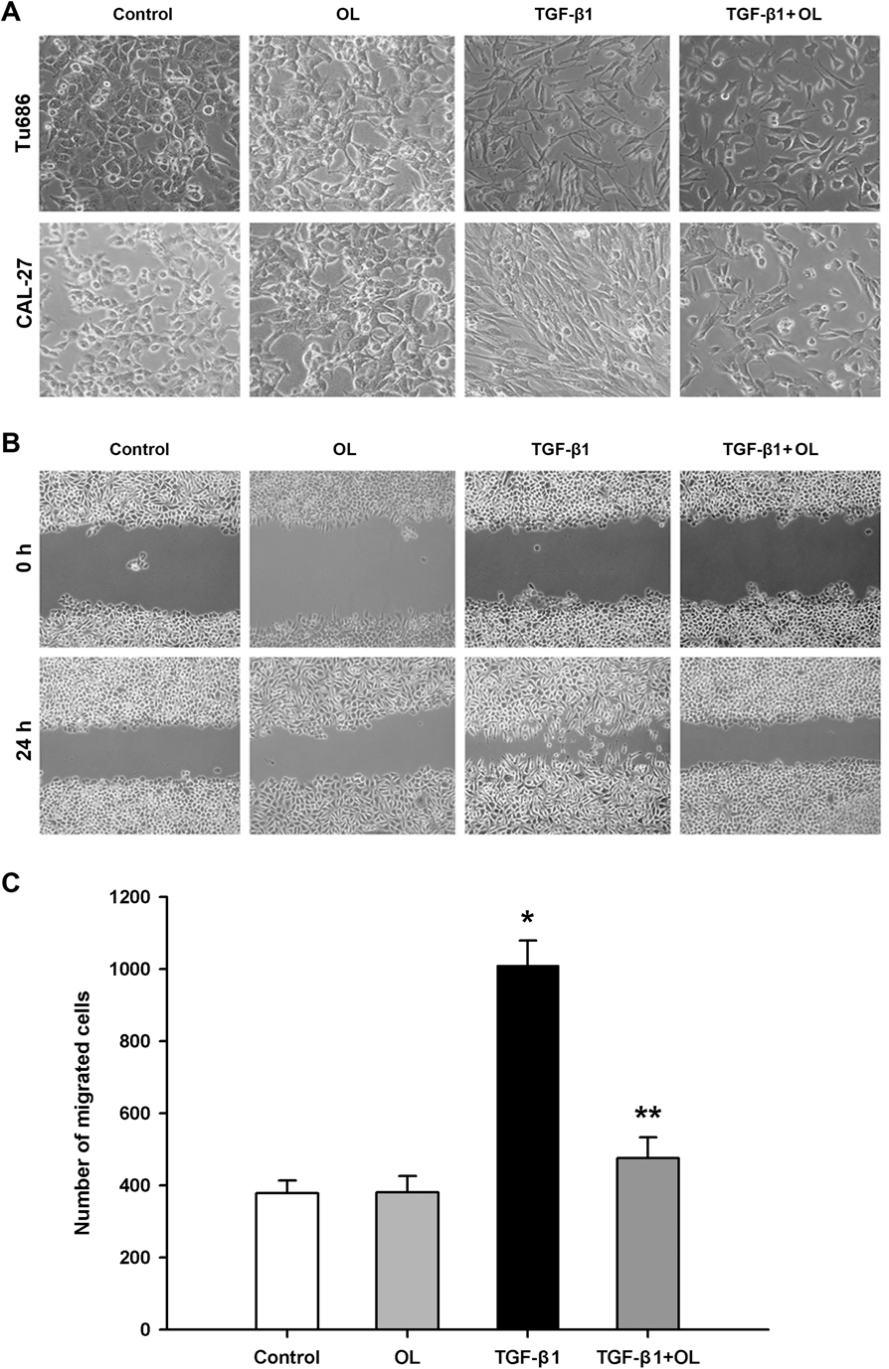
OL reversed the EMT and migration of Tu686 and CAL-27 cells induced by TGF-β1. **A** Morphological changes shown in OL and/or TGF-β1-treated Tu686 (up lane) and CAL-27 (down lane) cells. The cells were treated with PBS, 25 µg/mL OL, 10 ng/mL TGF-β1, and 10 ng/mL TGF-β1 + 25 µg/mL OL for 72 h, respectively. EMT changes were observed under microscope, ×100. **B**-**C** Wound-healing scratch assay photos and the statistical result for each group of Tu686 cells under the different treatments (at 24 h). The column chart shows the cell migration of each group of Tu686 cells at 24 h after treatment. *p <0.05 vs. control, and **p <0.05 vs. TGF-β1 group (tests were repeated for 3 times)

It was observed under the microscope that after treatment with 25 µg/mL of OL alone there was no obvious morphological change, but after the addition of 10 ng/mL of TGF-β1, both the Tu686 and CAL-27 cells showed obvious changes of EMT. The connection between the epithelium was loose and the antennae were extended, showing characteristic changes of EMT [18]. Nevertheless, after the addition of OL following TGF-β1, the antennae of stromal like cells became shorter and the cell arrangement was closer than that of the cells treated with TGF-β1 alone (Fig. 3A).

### OL attenuates the enhanced migration and invasion of Tu686 and CAL-27 cells induced by TGF-β1

**Fig. 4:**
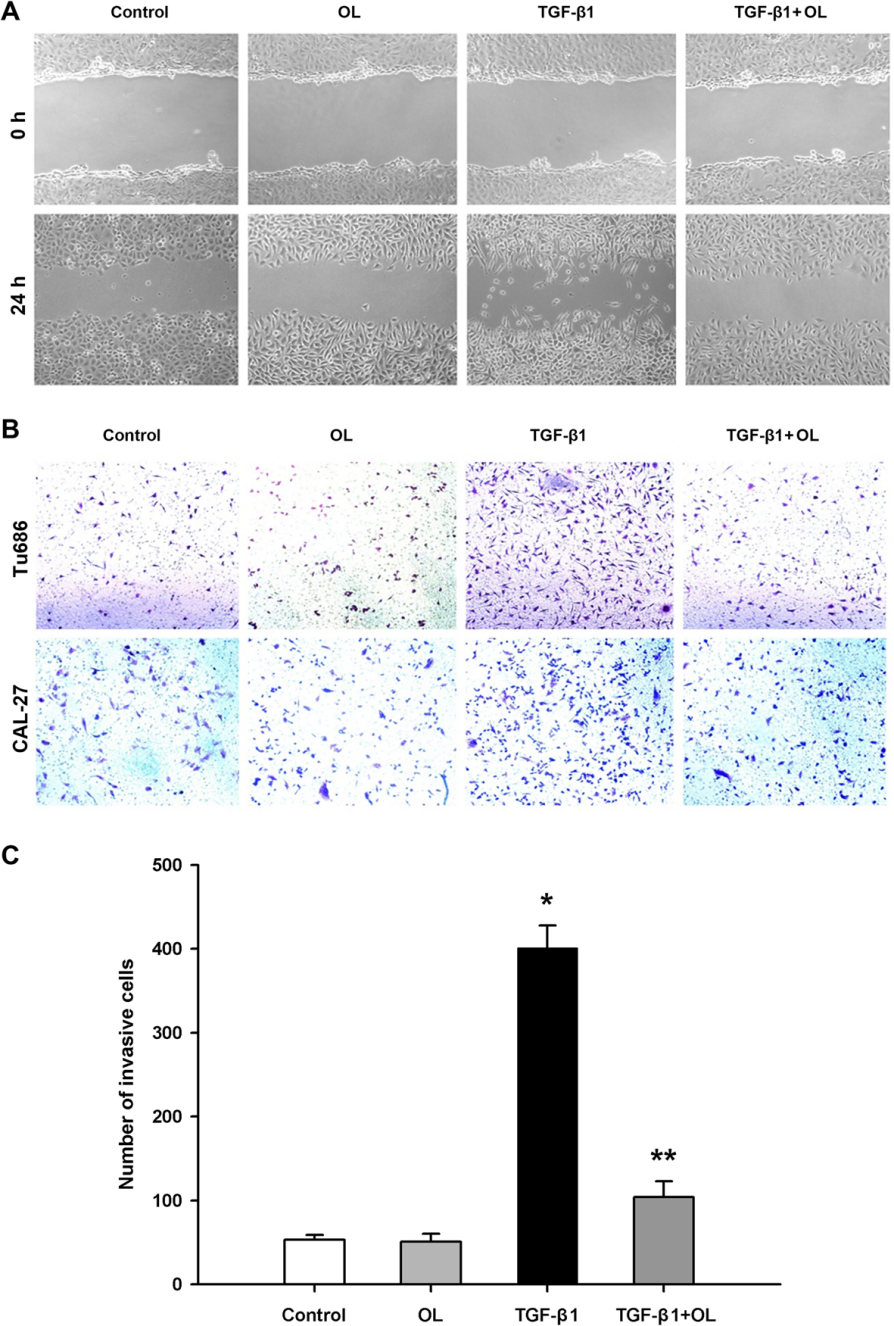
OL reversed the migration and invasion of Tu686 and CAL-27 cells induced by TGF-β1. **A** Wound-healing scratch assay photos were taken for each group of CAL-27 cells under the different treatments (at 0 h and 24 h). **B** Transwell assay photos were taken for each group of Tu686 (up lane) and CAL-27 (down lane) cells at 24 h after treatment. **C** Statistical column chart shows the cell invasive ability of each treatment group of Tu686 cells at 24 h after treatment. *p <0.05 vs. control, and **p <0.05 vs. TGF-β1 group (tests were repeated for 3 times)

As shown in Fig. 3B-C (*n* = 3–6 for each group), stimulation with a dose of 10 ng/mL of TGF-β1 could greatly enhance the migration ability of Tu686 cells. However, after treatment with OL (25 µg/mL) for 24 h, it was observed that the enhancement of migration induced by TGF-β1 was significantly inhibited. In addition, similar phenomena could be observed in CAL-27 cell line (Fig. 4A). Results of the invasion ability assays also showed that TGF-β1 stimulation enhanced the invasion behavior of Tu686 and CAL-27 cells, which was attenuated by OL treatment (Fig. 4B-C; *n* = 3–6 for Tu686 groups).

### OL affects the expression of EMT-related proteins

**Fig. 5:**
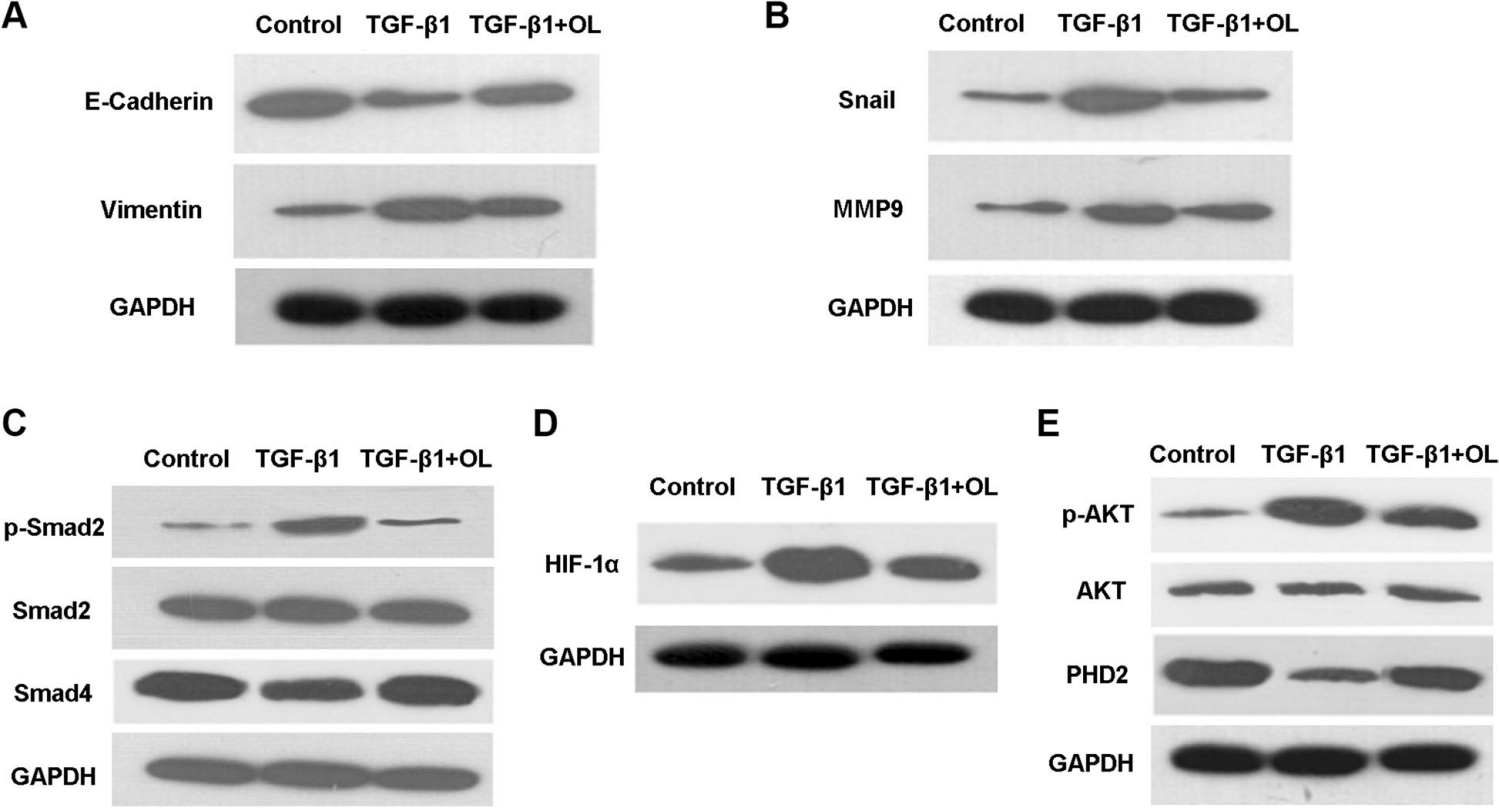
OL changed the levels of EMT-associated proteins and signaling pathways involved in TGF-β1-mediated invasion and metastasis in Tu686 cells. **A** Western blotting assay results about the E-cadherin and Vimentin expression in different groups (control, TGF-β1 and TGF-β1 + OL groups). **B** Western blotting results of Snail and MMP9 in different groups. Western blotting results showing that **C** TGF-β1 and OL affected the classical Smad2 signaling pathway, **D** HIF-1α expression, and **E** pVHL-dependent pathway and PI_3_K/AKT signaling proteins

After treatment with TGF-β1, the expression level of E-cadherin was significantly downregulated, and the levels of Vimentin, Snail and MMP9 were significantly increased. After the treatment with both TGF-β1 and OL, the decreased level of E-cadherin was significantly reversed, while the expression levels of Snail and MMP9 were significantly decreased compared with the TGF-β1 alone group (Fig. 5A-B).

### OL affects the classical TGF-β1-Smad2 and HIF-1α-related signaling pathways stimulated by TGF-β1

The phosphorylation level of Smad2 was significantly increased after the intervention of TGF-β1 (10 ng/mL), while this increase was significantly inhibited by the treatment of OL (25 µg/mL) (Fig. 5C). After adding OL (25 µg/mL), the increased level of HIF-1α induced by TGF-β1 (10 ng/mL) was significantly attenuated (Fig. 5D). The phosphorylation level of AKT was significantly increased after the intervention of TGF-β1, and it was significantly inhibited after the addition of OL. Moreover, the expression level of PDH2 significantly decreased after treatment with TGF-β1, while OL treatment partly reversed it (Fig. 5E).

### Effect observation of OL and TGF-β1 on Tu686 and CAL-27 cell lines

**Fig. 6:**
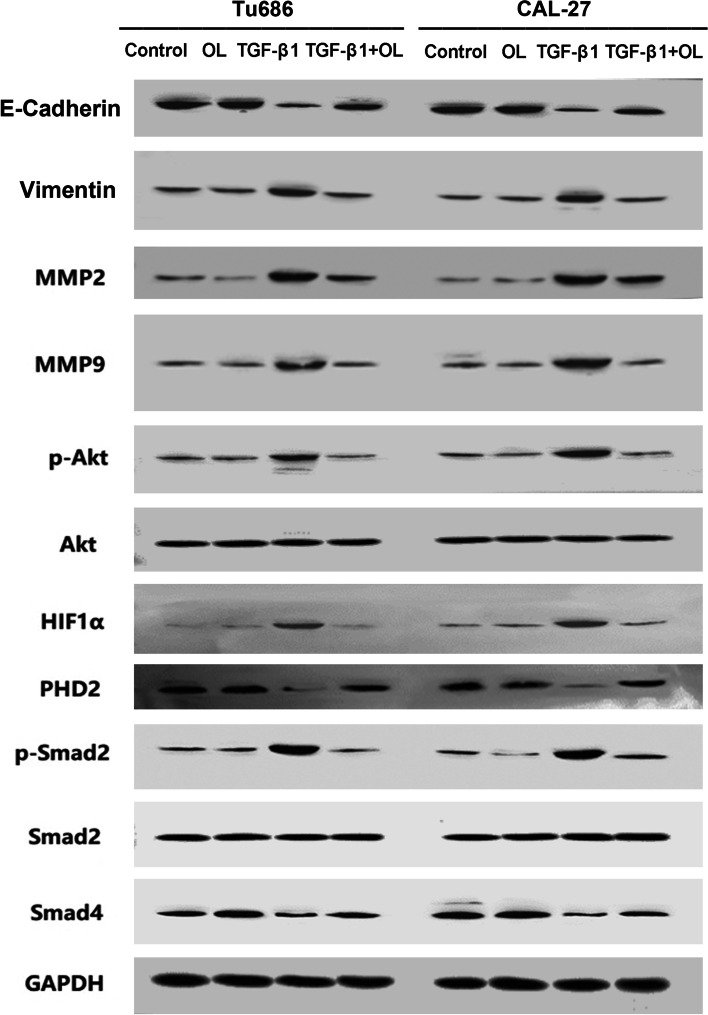
Effects of OL and TGF-β1 on EMT-related and TGF-β1-associated signaling proteins in Tu686 and CAL-27 cells. Left part, with representative result showing the influence of OL alone on the EMT-related and TGF-β1-associated signaling proteins in Tu686 cells. Right part, representative images showing influence of OL and TGF-β1 treatment on the EMT and TGF-β1 signaling-associated pathway proteins in CAL-27 cells

Representative images showing the effects of treatment with OL alone on the EMT-related proteins, the classical TGF-β1-Smad2 signaling protein, and HIF-1α-related signaling pathways in Tu686 cells, were presented in Fig. 6 (left part). In addition, representative images showing the influences of OL and TGF-β1 on the EMT-related proteins, the TGF-β1-Smad2 signaling, and the HIF-1α-related signaling pathways in CAL-27 cell line, were presented in Fig. 6 (right part). As shown here, OL alone had no obvious influence on the level of the detected proteins, and similar effects of OL and TGF-β1 treatment on these EMT-related and TGF-β1-associated signaling proteins in CAL-27 cells were also observed.

### OL inhibits the growth of SCCHN tumor in vivo

**Fig. 7:**
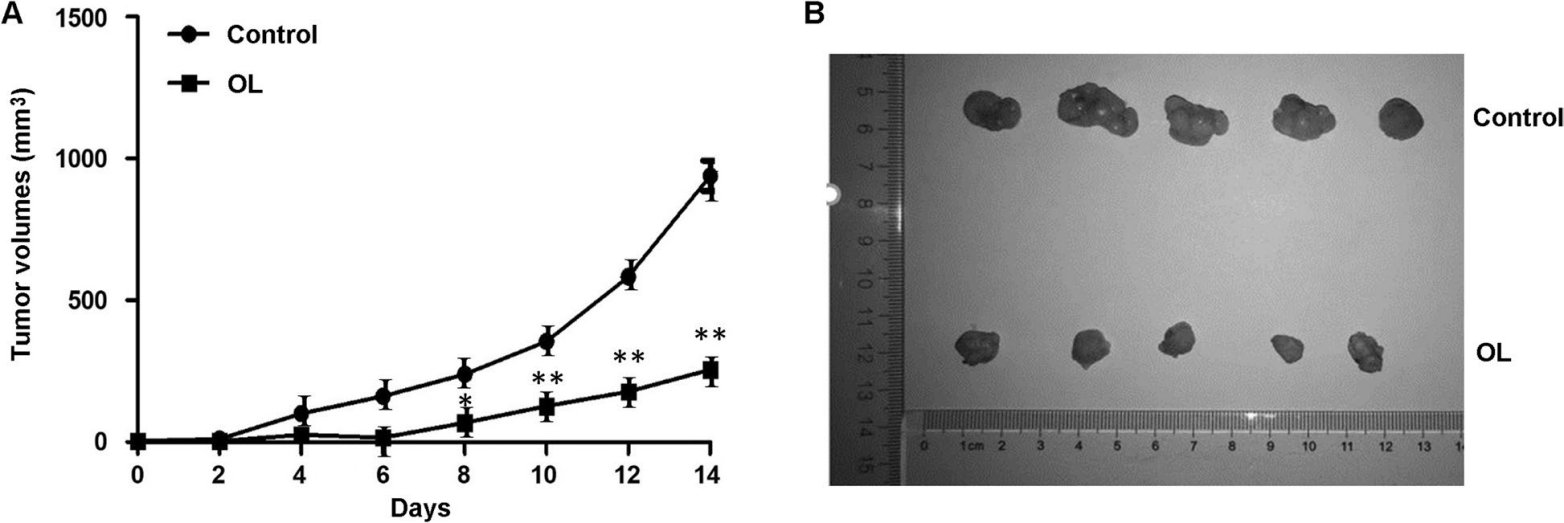
OL inhibited the growth of SCCHN xenograft tumor. M2 cells were treated with PBS (control group) and 1200 µg/mL OL (OL group) for 24 h, then the cells were subcutaneously injected into the submaxillary area of 7-week-old male nude mice (*n* = 5 for each group). **A** The xenograft tumor volumes were measured every two days and the growth curve was compiled (*p <0.05, **p <0.01 vs. control group). **B** The mice were sacrificed on the fourteenth day after transplantation, and the photos of the isolated xenograft tumors of the two groups were taken

The xenograft tumor-bearing mice injected with high metastasis cell line M2 cells, which were treated with PBS or OL (1200 µg/mL), were used for tumor growth observation for 14 days after transplantation. We found that the volume of xenograft tumors in the OL treatment group was significantly smaller when compared with that of the control group (Fig. 7A-B).

### OL alters the expression of EMT and metastasis-associated proteins in xenograft tumor model

**Fig. 8:**
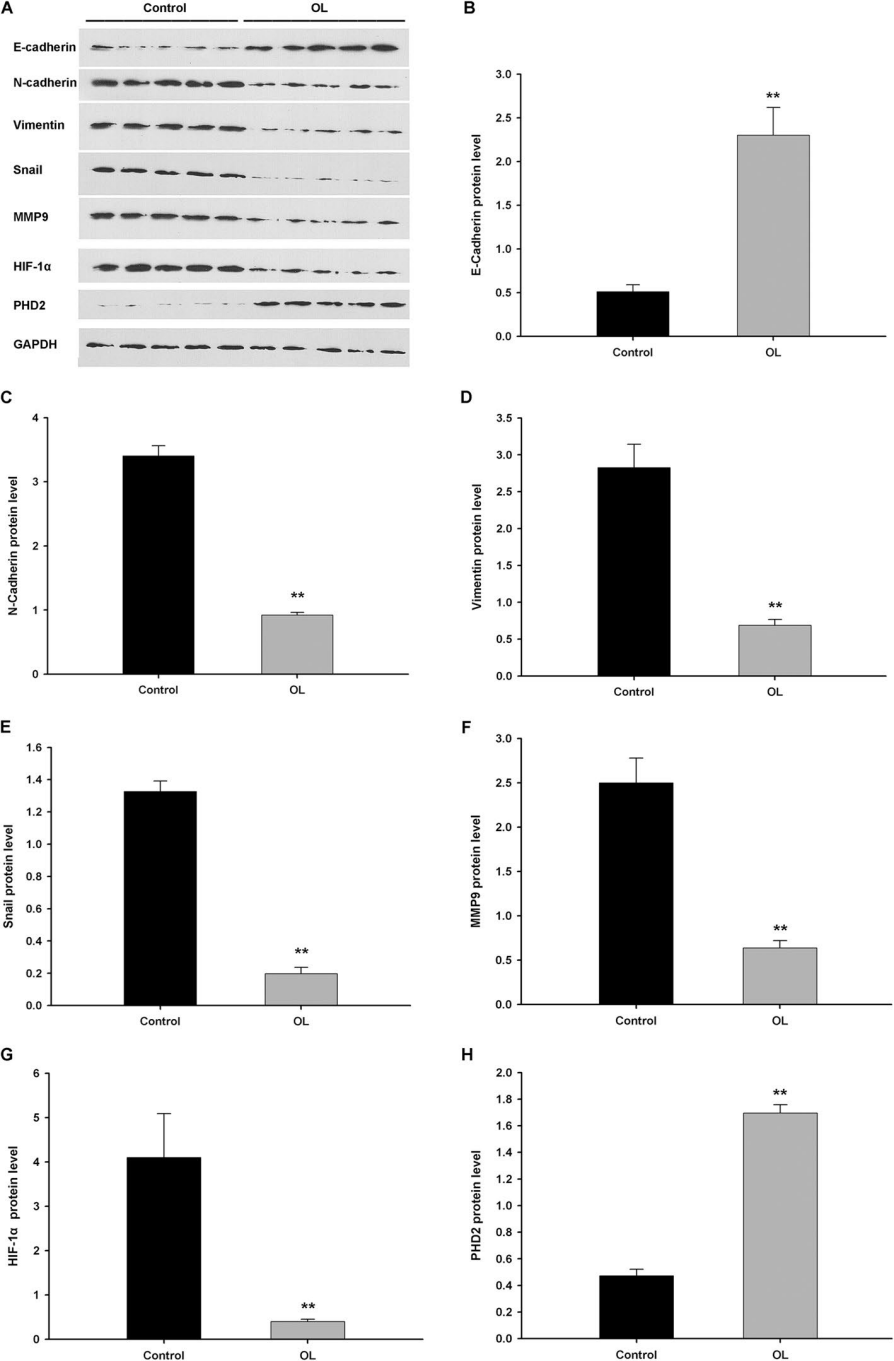
OL altered expression of EMT-associated proteins and HIF-1α pathway proteins in xenograft tumor. M2 cells were subcutaneously injected into submaxillary area of 7-week-old male nude mice (*n* = 5). After two weeks, the mice were sacrificed and the tumor tissue proteins were extracted for E-cadherin, N-cadherin, Vimentin, Snail, MMP9, HIF-1α and PHD2 analysis. **A** Representative Western blotting results of tumor tissue from 10 mice (*n* = 5 for each group) for the expression levels of the target proteins. The column diagrams show the expression levels of E-cadherin **B**, N-cadherin **C**, Vimentin **D**, Snail **E**, MMP9 **F**, HIF-1α **G** and PHD2 **H** of the two groups (**p <0.01 vs. control)

The expression levels of E-cadherin and PHD2 were significantly increased in the xenograft tumor tissues from mice injected with M2 cells and treated with OL, when compared with the results from the control group. The expression levels of N-cadherin, Vimentin, Snail, MMP9 and HIF-1α were significantly decreased in the xenograft tumor tissues from mouse model injected with M2 cells treated with OL, when compared with those of the control group (Fig. 8A-H).

## Discussion

Previous studies have shown that OL demonstrated anti-cancer effects in various types of tumors [19]. The prognosis is commonly poor when metastasis occurred in SCCHN patients. For those elder or advanced patients with metastasis, who have missed appropriate opportunity for surgery, radiotherapy and chemotherapy are deemed to be the most optimal treatment approach. Therefore, it is of particular importance to develop novel agents with favorable anticancer effect and tolerable toxicity and side effects. As a natural compound, OL shows low toxicity to human body, thus the prognosis of patients may be significantly improved when it could be applied for adjuvant chemotherapy in the future. However, the effect of OL on SCCHN has not been reported yet. In this study, a suitable non-toxic dose range of OL was screened through MTT test. The results were similar to the peritoneal dialysis test conducted by Lupinaccis et al. [11]. After treatment with 25 µg/mL of OL, the apoptosis rate of Tu686 cells was significantly increased, which indicated that OL may effectively induce apoptosis of SCCHN cells in vitro, and this finding is consistent with the antitumor effect of OL in other tumors.

Inhibition of metastasis is considered to be in the top priority to improve the prognosis of SCCHN. TGF-β1 can induce EMT alterations in Tu686 cells and promote cell invasion in vitro [7]. When OL intervention was added in this study, we observed that the change of morphology of Tu686 cells was reversed, with morphological changes similar to MET. Morphologically, cells in TGF-β1 + OL group were similar with that of the control group, and this finding was consistent with the report of Lupinaccis et al*.* [11]. This indicates that the effect of OL on reversing EMT is stable either in the process of promoting fibrosis or tumor metastasis. Scratch and Transwell assays verified that OL could reverse TGF-β1-induced migration and invasion of Tu686 cells. When the EMT process is activated, the expression of epithelial cell marker protein E-cadherin decreases, whereas the expression of interstitial cell marker proteins such as N-cadherin and Vimentin increases [20]. Many transcription factors, especially the Snail family, mediate the process of EMT. MMP9, one of the matrix metalloproteinases and most commonly involved in the EMT of SCCHN, can promote tumor invasion and metastasis by degrading extracellular matrix (ECM). At present, several studies were conducted about the inhibitory effects on tumor invasion and metastasis of OL or its related compounds in breast cancer, colon cancer and melanoma [12, 21, 22]. Among them, OL inhibits invasion by regulating EMT in MCF-7 cells of breast cancer. Our study showed that when OL was added, TGF-β1-induced changes of E-cadherin, Vimentin, Snail and MMP-9 were partially reversed, which further confirmed that OL could inhibit the invasion and migration of Tu686 cells in vitro.

**Fig. 9:**
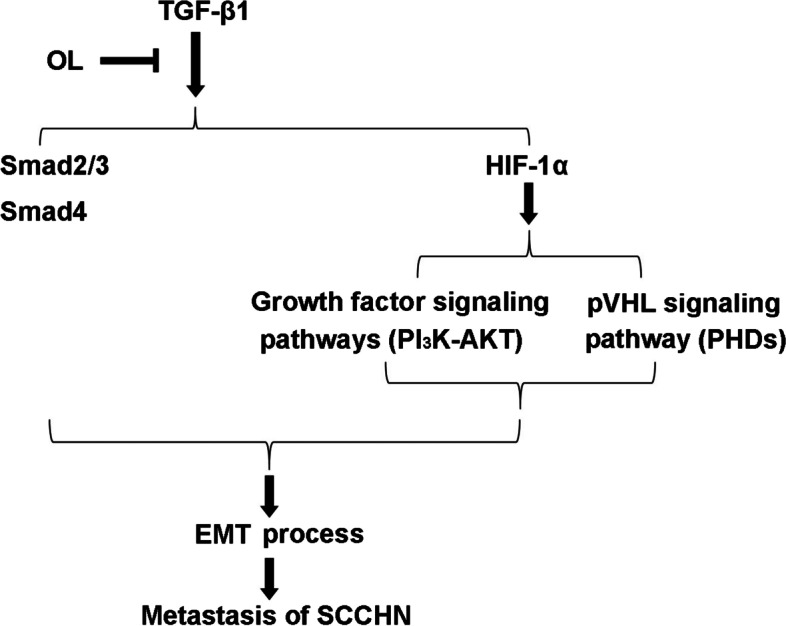
Schematic diagram showing the signaling pathways involved in the inhibitory effect of OL on EMT of SCCHN

We further detected the classical signal pathway involved in TGF-β1-induced EMT, Smad2/3 signal pathway. The phosphorylation level of Smad2 decreased significantly in TGF-β1 + OL group when compared with that of the TGF-β1 group. A previous study showed that OL inhibits the proliferation of colorectal cancer cells through downregulation of HIF-1α [13]. Our previous study also revealed that HIF-1α played an important regulatory role in OL-enhanced radiosensitivity of nasopharyngeal carcinoma [23]. HIF-1α, the principal regulator of hypoxia, mediates a variety of biological processes in tumor cells, including EMT [24]. In this study, TGF-β1 significantly increased HIF-1α expression in Tu686 cells, whereas the HIF-1α level was decreased by OL. There are multiple signaling pathways involved in HIF-1α regulation, including oxygen-dependent and oxygen-independent pathways. In this study, we detected the activation levels of PHD2 and AKT/pAKT which are involved in pVHL-dependent pathway and growth factor signaling pathway, respectively. The results showed that TGF-β1 significantly decreased PHD2 expression and OL partially reversed it. As we know, PHDs is an upstream regulator of HIF-1α, and its expression can be downregulated by hypoxia and consequently the degradation of HIF-1α is reduced [25]. In this study, TGF-β1 intervention alone rather than hypoxic environment can also affect the expression of PHD2, and this result was similar to a study reported in 2013, which suggests that TGF-β1 decreases PHD2 expression via a Smad-dependent signaling pathway, thereby leading to HIF-1α accumulation and EMT in renal tubular cells [26]. Those demonstrated that PHDs can act through oxygen-independent pathway. Meanwhile, OL is able to reverse TGF-β1/PHD2/HIF-1α signaling pathway. PI_3_K/AKT signal is involved in the growth factor signaling pathways. We found that TGF-β1 significantly increased the phosphorylation of AKT, which was effectively inhibited by OL. These findings suggest that OL can regulate the process of EMT by regulating the expression of HIF-1α. There are some complicated cross-talks between TGF-β1 signal, HIF-1α signal and PI_3_K/AKT signal, which need further investigation. The schematic diagram of OL intervention in TGF-β1 signaling pathway is shown in Fig. 9.

In order to further clarify the effect of OL on SCCHN, subcutaneous xenograft model of SCCHN cancer cells in nude mice was established. Advanced metastatic SCCHN cell line M2 was applied in this study. The tumor volume in OL group (mice injected with cells pretreated with OL) was smaller than that of the control group, indicating that OL has significant antitumor effect in vivo. In the verification of metastatic capacity of tumor cells, proteins in tumor tissues were extracted to detect the expression of EMT-related proteins. OL can significantly enhance the expression of epithelial cell marker E-cadherin, and reduce the expression of interstitial cell markers Vimentin, N-cadherin, and EMT-related transcription factor snail, indicating that OL effectively intervened the process of EMT in vivo. The change of MMP9 suggests that OL could inhibit the invasion and metastasis of SCCHN tumor cells. In addition, we also detected the levels of HIF-1α and PHD2 in tissues of these two groups. OL significantly decreased the level of HIF-1α, but increased the expression of PHD2, showing the expression level of HIF-1α was negatively correlated with PHD2. These findings show that HIF-1α signaling may be involved in the inhibitory effect of OL on tumor metastasis in vivo.

In conclusion, this study preliminarily clarified that OL can inhibit the process of EMT, invasion and metastasis of SCCHN cells in vitro and in vivo. The findings of this study provide a basis for the application of the natural compound OL in the treatment of SCCHN in the future. The comprehensive mechanisms involved need to be further investigated.

## Supplementary Information

**Additional file 1.**

## Data Availability

All data generated or analyzed during this study are included in this published article.
