# Supplementary material for: Oleuropein inhibits invasion of squamous cell carcinoma of the head and neck through TGF-β1 signaling pathway
Source: BMC Cancer. 2022 Sep 1;22:942. doi: 10.1186/s12885-022-09979-2 (PMC9434901; doi:10.1186/s12885-022-09979-2)
Supplement: Supplementary file 1 — Additional file 1. [file 12885_2022_9979_MOESM1_ESM.docx]

**Oleuropein inhibits invasion of squamous cell carcinoma of the head and neck through TGF-β1 signaling pathway**

Ting Xu, Xuan Liu

Running title: Oleuropein inhibits head and neck cancer

**Supplementary Figure**


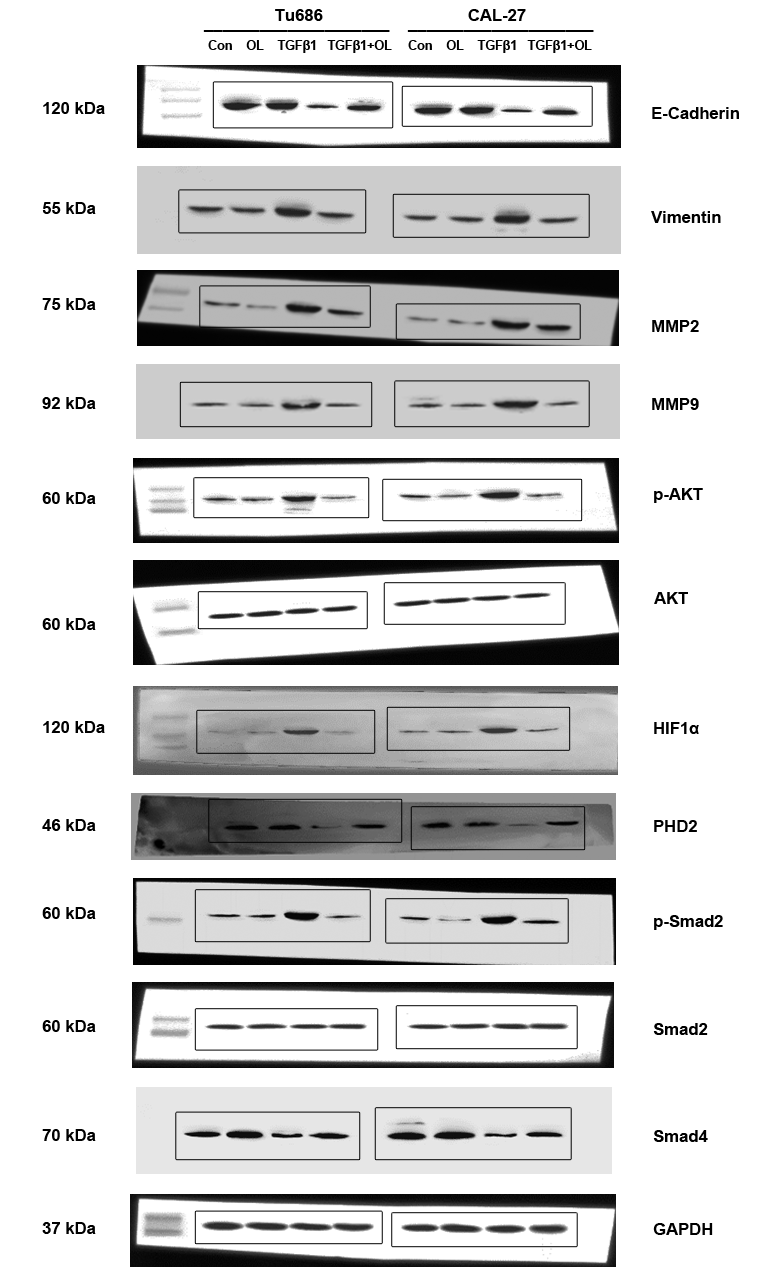


**Supplementary Figure 1.** Full-length original images of Western blots showing the effects of OL and TGF-β1 on EMT-related and TGF-β1-associated signaling proteins in Tu686 and CAL-27 cells (in four groups: Control, OL, TGF-β1, TGF-β1+OL). These detected protein blots were corresponding to the presented blots in Figure 4 in the same order and locations. The blots marked with boxes in the left part denoting these proteins detected with samples from the OL or/and TGF-β1 treated Tu686 cells, and the right part the CAL-27 cells.
